# Clinical Evaluation of Solitary Median Maxillary Central Incisor Syndrome

**DOI:** 10.1155/2019/2637825

**Published:** 2019-09-12

**Authors:** Malaz M. Mustafa, M. Zakirulla, Ibrahim AlShahrani, Rafi A. Togoo, Zuhair M. Alkahtani, Tasneem S. Ain

**Affiliations:** Department of Pediatric Dentistry & Orthodontic Sciences, College of Dentistry, King Khalid University, Abha, Saudi Arabia

## Abstract

Solitary median maxillary central incisor (SMMCI) is a rare dental anomaly. It is estimated to occur in 1 : 50,000 live births. The SMMCI tooth differs from the normal central incisor in that the crown form is symmetric and it develops and erupts precisely in the midline of the maxillary dental arch in both primary and permanent dentitions. The presence of SMMCI with hemifacial microsomia (HFM) is a very rare clinical condition. We report a case of SMMCI in a female of African ethnic origin, who presented with SMMCI in permanent dentition with mild nasal stenosis. An early diagnosis of SMMCI is important, since it may be a sign for other severe congenital or developmental abnormalities. Therefore, systematic follow-up and close monitoring of the growth and development of SMMCI patients are crucial.

## 1. Introduction

A solitary median maxillary central incisor (SMMCI) is a rare malformation associated with defects of midline structures, including the craniofacial bones, nasal airways (choanal atresia and nasal pyriform aperture stenosis), and developing brain (holoprosencephaly [HPE]), along with an increased risk of pituitary malformation and malfunction [1]. A large number of midline developmental defects, such as hypotelorism (closely spaced eyes) and microcephaly (small head circumference), have been reported in patients with SMMCI [2]. Sex predominance in SMMCI is a debatable issue, with some studies stating a female predominance, while others not stating any sex predilection [3, 4]. The etiology of SMMCI is unknown, but it may be related to a disruption in the development of the maxilla, which occurs during gestation at approximately 35 to 38 days, with abnormal formation of tooth germs and alveolar bone. The most common clinical characteristic of this malformation in the oral cavity is the presence of a single central incisor in the midline of the maxilla in both the dentitions [5].

The name originally given to this syndrome by Hall et al. [1] was “solitary median maxillary central incisor, short stature, choanal atresia/midnasal stenosis syndrome.” It has now been customarily shortened to “solitary median maxillary central incisor syndrome” or SMMCI syndrome.

A family history of SMMCI or of holoprosencephaly, slow learning or intellectual disability, congenital nasal obstruction, microcephaly, epilepsy, very short stature, or other midline defects are seen in 25% of the cases [2, 6, 7]. Furthermore, studies have confirmed the suggestion that congenital nasal pyriform aperture stenosis (CNPAS) probably represents a manifestation of HPE [8]. SMMCI is detected in approximately 60% of CNPAS cases. The purpose of this paper was to report a case of a patient with a SMMCI.

## 2. Case Report

A 12-year-old female of African ethnic origin was reported to the pediatric dental clinic of King Khalid University, College of Dentistry. She was the third child among her five siblings, with no history of hereditary diseases reported by the accompanying mother. The birth history of the patient was uneventful and was born as a full-term neonate of average birth weight by spontaneous normal delivery to healthy, nonconsanguineous parents. However, soon after birth, she suffered breathing difficulties and respiratory distress that necessitated her hospital admission for 37 days under observation. A diagnosis of mild nasal stenosis was made that did not require any intervention or treatment. The patient was then discharged, but there was no follow-up due to the patient's failure to appear for follow-up visits.

**Figure 1:**
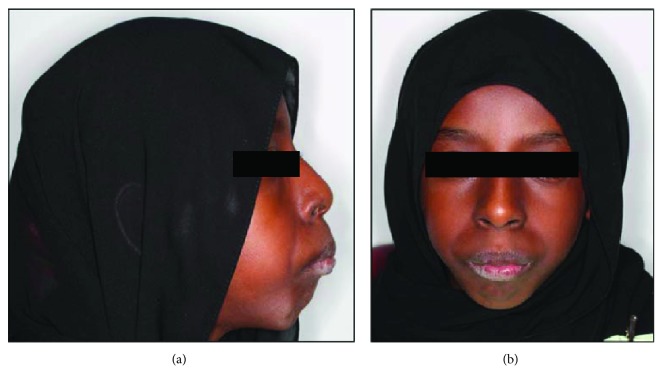
Extraoral photograph of the patient showing (a) convex lateral facial profile and (b) frontal facial view where prominent nasal bone, narrow nose, indistinctive philtrum, and incompetent lips can be seen.

**Figure 2:**
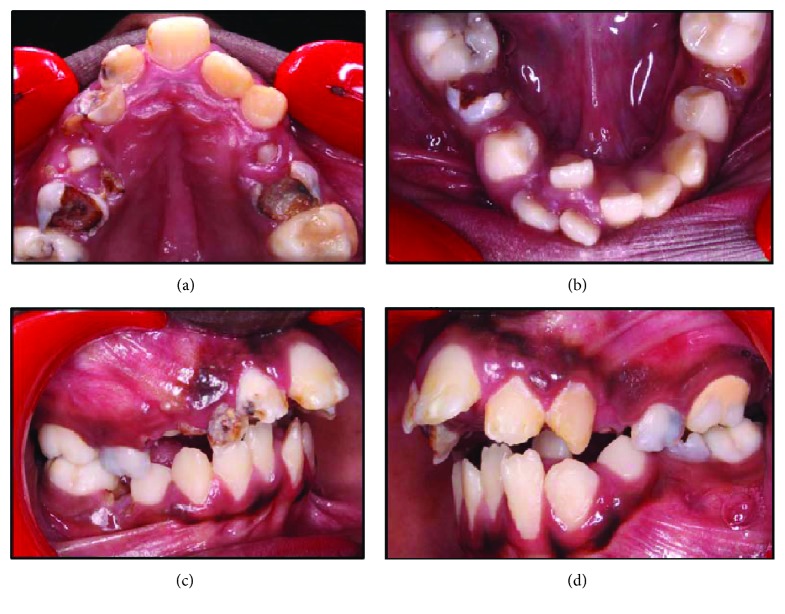
Intraoral photographs are showing (a) upper jaw with characteristic prominent median palatal suture, (b) lower jaw with lingually erupting #42 indicating crowding; (c) right side view is showing sinus tract related to #54; and (2d) left side view showing heavy calculus accumulation.

On extraoral examination, the patient had a noticeable convex facial profile, poorly distinctive philtrum of the upper lip, prominent nasal bone, narrow nose, and obviously incompetent lips (Figure 1). Intraorally, the maxillary labial frenum was entirely absent, and the palate was narrow, high arched, and V-shaped with a prominent midpalatal suture (Figure 2(a)). The dentition was in the mixed dentition stage, with Angle's quarter cusp class II molar occlusal relation on the right side, while the left side indicated a full cusp class II relation. An extreme horizontal overbite (>7 mm) was noted. Rampant caries was evident with retained root stumps of primary molars. On the left side, the maxillary and mandibular teeth exhibited heavy plaque and calculus accumulation as compared to the right, indicating a right-side chewing dominance. No abnormality was detected with respect to the oral mucosa and gingival tissues, except for a small sinus tract related to the remaining root stumps of the maxillary right first primary molar (Figures 2(b)–2(d)).

**Figure 3:**
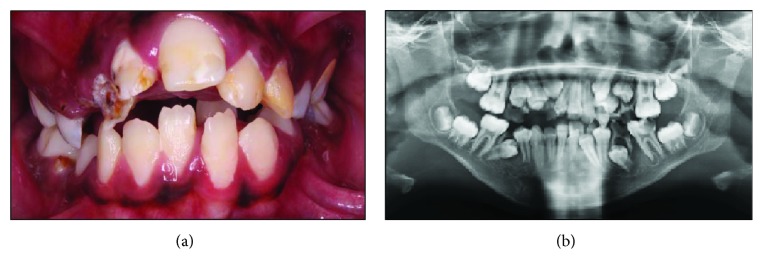
Images show (a) an intraoral image showing the frontal view of SMMCI and (b) orthopantomography (OPG) showing SMMCI.

**Figure 4:**
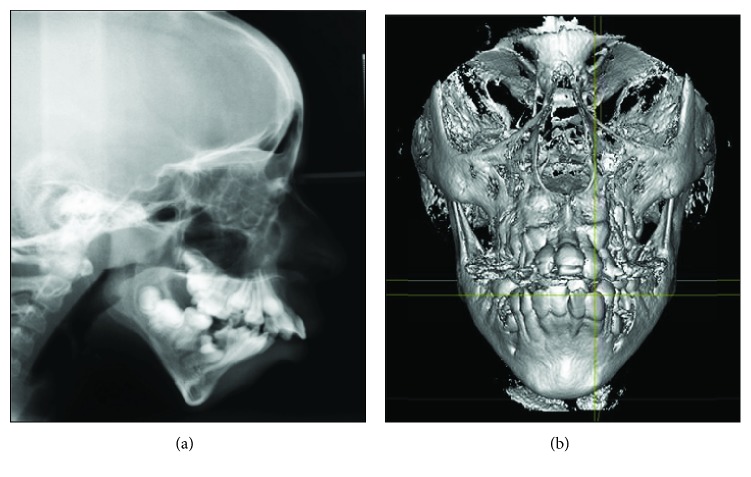
Images shows (a) lateral cephalogram and (b) cone beam computed tomography (CBCT) frontal view showing SMMCI.

An SMMCI was present precisely at the midline, and the patient's mother reported its presence in the primary dentition as well. It exhibited mirror image symmetry between its right and left sides, which mimicked the anatomic contour of the distal surface of a normal maxillary central incisor (Figure 3). The patient was referred for orthodontic evaluation and assessment. A true lateral view (lateral cephalogram) of the patient revealed a class II skeletal pattern with a downward and backward mandibular rotation. Both Anterior Nasal Spine (ANS) and Sella Nasion-Mandibular Plane (SNMP) angles showed a huge disparity from normal values, which reflected a highly prominent premaxilla (Figure 4(a)). The child was later referred to a pediatrician for the assessment of her overall physical status and for further investigation. The patient was categorized as underweight as her body mass index (BMI) at the time of consultation was 13.4, which put her below the fifth percentile in the growth chart. No abnormalities were detected in the child's intelligence quotient level, brain structure, or growth hormone levels. However, nasal stenosis was observed on frontal cone beam computed tomography (Figure 4(b)).

The treatment plan for the current case consisted of two main phases: a preparatory phase of comprehensive oral rehabilitation of dental diseases, including extraction of all residual roots of primary molars and restoration of carious permanent teeth, followed by a corrective phase that would involve complex orthodontic treatment.

## 3. Discussion

SMMCI is a rare developmental anomaly that may occur as an isolated dental disorder or may be a part of a syndrome accompanied by a range of midline field defects [9]. The SMMCI syndrome is a complex syndrome comprising various defects, mainly midline defects of development that result from unknown factor(s) operating in utero around the 35th to 38th day from conception [1]. Although the etiology is unknown, it is believed to have a genetic background involving a mutation in gene Sonic Hedgehog (SHH) in chromosome 7q36.1 [10]. SMMCI has been reported to occur due to the fusion of two deciduous tooth germs and the permanent maxillary central incisors [11]. The single central incisor erupts exactly in the midaxial region and differs in morphology from the normal central incisors in that it exhibits a symmetrical dental crown (as seen in the present case report where an SMMCI was located precisely in the midline) with identical right and left surfaces, unlike a normal central incisor [2, 12].

The condition of SMMCI can be recognized prior to the eruption of the deciduous single incisor. Diagnosis can be made prenatally with ultrasound or genetic testing; however, it is more commonly confirmed at birth. The child may present a notched upper lip, along with the absence of a labial frenulum and a narrow nose. Preterm birth and low birth weight are also seen in 37% of the cases [1]. In the present case, the patient was a full-term neonate with average birth weight, as reported by her mother.

In the literature, more than 70 systemic abnormalities have been described in SMMCI patients without a defined syndrome [13, 14]. SMMCI may occur as an isolated trade or along with various other midline developmental defects including “congenital heart disease” [15]; “short stature” [14–18]; “congenital nasal pyriform aperture stenosis” [19, 20]; “pituitary insufficiency” [17, 18]; “microcephaly” [15]; “midnasal stenosis” [16]; “scoliosis” [15]; choanal atresia; cleft lip and cleft palate; less commonly microcephaly, hypopituitarism, hypotelorism, convergent strabismus, esophageal and duodenal atresia, cervical hemivertebrae, cervical dermoid, hypothyroidism, scoliosis, absent kidney, micropenis, and ambiguous genitalia. 50% of the reported cases exhibited short stature [14, 16]. On the other hand, numerous identified syndromes have been reported in SMMCI patients, including ectodermal dysplasia [21], Duane retraction syndrome [22], velocardiofacial syndrome, CHARGE syndrome, vacterl associations, and HPE. Systemic conditions were found in 50% of cases including 20% asthma and allergies; multiple hemangiomas; alopecia with parchment skin; ptosis; ocular coloboma; congenital talipes equinovarus; oligodontia; and absent thumb [16]. Less common manifestations comprise diabetic pregnancies (14%), preterm labor, and low birth weight (37%) [1].

In our case report, the patient was diagnosed with mild nasal stenosis since birth. Congenital nasal malformation associated with SMMCI (midnasal stenosis, choanal atresia, or congenital narrowing of nasal pyriform aperture). The patient's mother reported that the patient suffered severe respiratory distress soon after birth for which she was hospitalized for approximately 37 days. Previous studies have reported that respiratory problems are quite frequent among SMMCI cases due to nasal stenosis, which makes it difficult for newborn babies to breathe through the nostrils. The presence of an SMMCI tooth can be an indicator of associated anomalies, particularly of the serious anomaly of holoprosencephaly [23].

On extraoral examination, the patient presented a noticeable convex facial profile, poorly distinctive philtrum of the upper lip, prominent nasal bone, narrow nose, and obvious incompetent lips. Previous cases have been reported wherein SMMCI was associated with features such as pseudo-notched or arch-shaped upper lip appearance with an indistinct philtrum, which is believed to be due to the prominent maxillary alveolus over the developing primary SMMCI tooth. Other features include nonexistence of the labial frenulum, with a narrow nose, a V-shaped palate, and narrow ridge along the midpalatal suture.

In several studies conducted earlier, SMMCI syndrome has been associated with a short stature and growth hormone deficiency [7, 11, 24]. The patient in this report was found to be underweight as per her BMI. Dental experts are familiar with the fundamentals of SMMCI syndrome and can help detect serious associated abnormalities such as HPE. Thus, they can suggest proper referral or treatment to the affected patients. Bolan et al. rightly mention that dentists are usually the first health professionals to face patients with SMMCI, which makes it obligatory for them to know and understand the appropriate diagnosis and treatment plan [25]. Various treatment options can be adopted for treating SMMCI cases depending upon its severity and associated problems.

Proper management of SMMCI (following diagnosis and genetic counseling) necessitates comprehensive pediatric dental care if the case presentation included SMMCI and mild nasal airway narrowing exclusively without other manifestations. A facial growth pattern in both transverse and sagittal directions should be analyzed in addition to serial photographs taken as part of routine dental reviews. Intervention is not required in the primary dentition [14]. In the permanent dentition stage, orthodontic intervention will be in the form of palatal expansion to accommodate sufficient space for the SMMCI and growing maxilla. Furthermore, a space required to accommodate for a contralateral artificial central incisor can be provided by either distalization of the first permanent molars or, otherwise, extraction of premolars. Artificial replacement of the contralateral missing central incisor can be in the form of a spoon denture, bridge, or single tooth implant at an older age (17-18 years old) [1]. Some cases might require extraction of the SMMCI followed by mesialization and reshaping of the laterals, canines, and premolars [12]. In case of associated syndromes, a more complex treatment requiring interdisciplinary care might be necessary [14].

## 4. Conclusion

A multidisciplinary approach is essential for the management of affected individuals and their families. Early diagnosis of SMMCI is important, as it may be a sign of other severe congenital or developmental abnormalities. Therefore, systematic follow-up and close monitoring of the growth and development of SMMCI patients are crucial. Referral to a pediatrician for further investigation is important. Comprehensive dental management for the patient should be tailored to suit the patient's need. Medical input needs to be supplemented by a team including a pedodontist, an orthodontist, an endodontist, and an oral surgeon, as well as a speech therapist and a psychologist.
